# Tetra­carbon­yl[bis­(diphenyl­phosphan­yl)tetra­methyl­disiloxane-κ^2^ *P*,*P*′]chromium(0)

**DOI:** 10.1107/S1600536812000219

**Published:** 2012-01-07

**Authors:** Normen Peulecke, Bernd H. Müller, Anke Spannenberg, Uwe Rosenthal

**Affiliations:** a Leibniz-Institut für Katalyse e. V. an der Universität Rostock, Albert-Einstein-Strasse 29a, 18059 Rostock, Germany

## Abstract

The title compound, [Cr(C_28_H_32_OP_2_Si_2_)(CO)_4_], was obtained by the ligand-exchange reaction of Cr(CO)_6_ with (Ph_2_PSiMe_2_)_2_O in refluxing toluene. The CrC_4_P_2_ coordination geometry is distorted octa­hedral, with a P—Cr—P bite angle of 99.22 (4)°.

## Related literature

For the synthesis of (Ph_2_PSiMe_2_)_2_O, using (SiMe_2_Cl)_2_O instead of SiMe_2_Cl_2_, see: Hassler & Seidl (1988 ▶). For the structures of complexes of group III metals with (H_2_PSi^i^Pr_2_)_2_O, see: von Hänisch & Stahl (2006 ▶, 2007 ▶), and for group II metals, see: Kopecky *et al.* (2010 ▶). For the structure of a chromium complex with a silicon-bridged bis­phosphine, see: Peulecke *et al.* (2010 ▶).
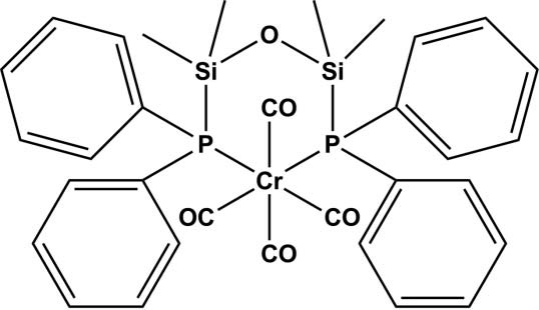


## Experimental

### 

#### Crystal data

[Cr(C_28_H_32_OP_2_Si_2_)(CO)_4_]
*M* *_r_* = 666.70
Monoclinic, 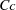

*a* = 9.2722 (5) Å
*b* = 21.4148 (15) Å
*c* = 16.5695 (8) Å
β = 95.200 (4)°
*V* = 3276.5 (3) Å^3^
*Z* = 4
Mo *K*α radiation
μ = 0.56 mm^−1^
*T* = 150 K
0.30 × 0.21 × 0.12 mm

#### Data collection

Stoe IPDS II diffractometer
Absorption correction: numerical (*X-SHAPE* and *X-RED32*; Stoe & Cie, 2005 ▶) *T*_min_ = 0.830, *T*_max_ = 0.952
21143 measured reflections
5756 independent reflections
4303 reflections with *I* > 2σ(*I*)
*R*_int_ = 0.071

#### Refinement

*R*[*F* ^2^ > 2σ(*F* ^2^)] = 0.035
*wR*(*F* ^2^) = 0.059
*S* = 0.79
5756 reflections
335 parameters
2 restraints
H-atom parameters constrained
Δρ_max_ = 0.32 e Å^−3^
Δρ_min_ = −0.20 e Å^−3^
Absolute structure: Flack (1983 ▶), 2799 Friedel pairs
Flack parameter: −0.021 (19)

### 

Data collection: *X-AREA* (Stoe & Cie, 2005 ▶); cell refinement: *X-AREA*; data reduction: *X-AREA*; program(s) used to solve structure: *SHELXS97* (Sheldrick, 2008 ▶); program(s) used to refine structure: *SHELXL97* (Sheldrick, 2008 ▶); molecular graphics: *XP* in *SHELXTL* (Sheldrick, 2008 ▶); software used to prepare material for publication: *SHELXL97*.

## Supplementary Material

Crystal structure: contains datablock(s) I, global. DOI: 10.1107/S1600536812000219/yk2038sup1.cif

Structure factors: contains datablock(s) I. DOI: 10.1107/S1600536812000219/yk2038Isup2.hkl

Additional supplementary materials:  crystallographic information; 3D view; checkCIF report

## Figures and Tables

**Fig. 1.**
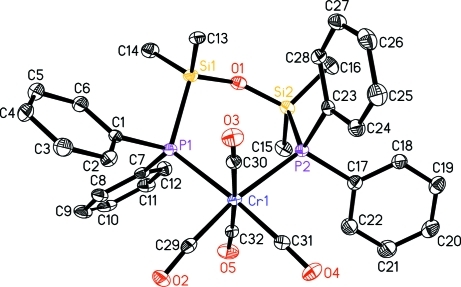
The molecular structure of the title compound with atom labels and 30% displacement ellipsoids. Hydrogen atoms are omitted for clarity.

## supplementary crystallographic information

### Comment

Diphosphines (like diphenylphosphanylpropane) are widely used as chelating ligands for the complex formation. Among them the siloxane-bridged ligands and their complexes with transition metals are not known. The few examples were made for (H_2_PSi^i^Pr_2_)_2_O coordinated with Al, Ga or In yielding polycyclic structures (von Hänisch *et al.*, 2006, 2007) or with Mg, Ca, Sr or Ba yielding only in the case of Mg a monomeric structure (Kopecky *et al.*, 2010). During our studies on the selective oligomerization of ethene *via* transition metal-catalyzed tri- or tetramerization we became interested in chromium complexes with ligands of this class (*e.g.* (Ph_2_P)_2_SiMe_2_: Peulecke *et al.*, 2010). In the present publication, we report the preparation and crystal structure of C_32_H_32_CrO_5_P_2_Si_2_, which was observed to be the single product of a reaction of (Ph_2_PSiMe_2_)_2_O with Cr(CO)_6_. The coordination geometry at the chromium centre is distorted octahedral. The observed bite-angle P—Cr—P is 99.22 (4)°.

### Experimental

Cr(CO)_6_ (175 mg, 0.8 mmol) was added to a solution of (Ph_2_PSiMe_2_)_2_O (321 mg, 0.75 mmol) in 20 ml toluene and the resulting mixture was stirred at reflux temperature for 72 h. Subsequently, the formed yellow solution was cooled down to 0°C and filtered. Toluene was removed and the product was extracted with dichloromethane. The major part of dichloromethane was removed and the remaining solution was over-layered with *n*-hexane to get single crystals of the title compound, which were suitable for X-ray crystal structure analysis. The pale yellow compound was fully characterized by standard analytical methods, ^31^P-NMR: (CD_2_Cl_2_): 5,9 p.p.m..

### Refinement

H atoms were placed in idealized positions with d(C—H) = 0.95 Å (CH) and 0.98 Å (CH_3_) and refined using a riding model with *U*_iso_(H) fixed at 1.2 *U*_eq_(C) for CH and 1.5 *U*_eq_(C) for CH_3_.

### Crystal data

**Table:**

[Cr(C_28_H_32_OP_2_Si_2_)(CO)_4_]	*F*(000) = 1384
*M**_r_* = 666.70	*D*_x_ = 1.352 Mg m^−^^3^
Monoclinic, *C**c*	Mo *K*α radiation, λ = 0.71073 Å
Hall symbol: C -2yc	Cell parameters from 4413 reflections
*a* = 9.2722 (5) Å	θ = 1.9–28.3°
*b* = 21.4148 (15) Å	µ = 0.56 mm^−^^1^
*c* = 16.5695 (8) Å	*T* = 150 K
β = 95.200 (4)°	Prism, yellow
*V* = 3276.5 (3) Å^3^	0.30 × 0.21 × 0.12 mm
*Z* = 4	

### Data collection

**Table:**

Stoe IPDS II diffractometer	5756 independent reflections
Radiation source: fine-focus sealed tube	4303 reflections with *I* > 2σ(*I*)
graphite	*R*_int_ = 0.071
ω scans	θ_max_ = 25.3°, θ_min_ = 1.9°
Absorption correction: numerical (*X-SHAPE* and *X-RED32*; Stoe & Cie, 2005)	*h* = −11→11
*T*_min_ = 0.830, *T*_max_ = 0.952	*k* = −25→25
21143 measured reflections	*l* = −19→19

### Refinement

**Table:**

Refinement on *F*^2^	Secondary atom site location: difference Fourier map
Least-squares matrix: full	Hydrogen site location: inferred from neighbouring sites
*R*[*F*^2^ > 2σ(*F*^2^)] = 0.035	H-atom parameters constrained
*wR*(*F*^2^) = 0.059	*w* = 1/[σ^2^(*F*_o_^2^) + (0.0178*P*)^2^] where *P* = (*F*_o_^2^ + 2*F*_c_^2^)/3
*S* = 0.79	(Δ/σ)_max_ < 0.001
5756 reflections	Δρ_max_ = 0.32 e Å^−^^3^
335 parameters	Δρ_min_ = −0.20 e Å^−^^3^
2 restraints	Absolute structure: Flack (1983), 2799 Friedel pairs
Primary atom site location: structure-invariant direct methods	Flack parameter: −0.021 (19)

### Special details

**Table:**

Geometry. All s.u.'s (except the s.u. in the dihedral angle between two l.s. planes) are estimated using the full covariance matrix. The cell s.u.'s are taken into account individually in the estimation of s.u.'s in distances, angles and torsion angles; correlations between s.u.'s in cell parameters are only used when they are defined by crystal symmetry. An approximate (isotropic) treatment of cell s.u.'s is used for estimating s.u.'s involving l.s. planes.
Refinement. Refinement of *F*^2^ against ALL reflections. The weighted *R*-factor *wR* and goodness of fit *S* are based on *F*^2^, conventional *R*-factors *R* are based on *F*, with *F* set to zero for negative *F*^2^. The threshold expression of *F*^2^ > σ(*F*^2^) is used only for calculating *R*-factors(gt) *etc*. and is not relevant to the choice of reflections for refinement. *R*-factors based on *F*^2^ are statistically about twice as large as those based on *F*, and *R*- factors based on ALL data will be even larger.

### Fractional atomic coordinates and isotropic or equivalent isotropic displacement parameters (Å^2^)

**Table:**

	*x*	*y*	*z*	*U*_iso_*/*U*_eq_	
C1	0.7971 (2)	0.47102 (10)	0.89997 (14)	0.0260 (8)	
C2	0.9455 (2)	0.46252 (10)	0.91594 (14)	0.0323 (9)	
H2	0.9816	0.4315	0.9534	0.039*	
C3	1.04124 (19)	0.49949 (13)	0.87708 (16)	0.0404 (11)	
H3	1.1427	0.4937	0.8880	0.048*	
C4	0.9885 (3)	0.54495 (12)	0.82225 (15)	0.0464 (12)	
H4	1.0539	0.5702	0.7957	0.056*	
C5	0.8400 (3)	0.55345 (11)	0.80628 (14)	0.0445 (11)	
H5	0.8039	0.5845	0.7688	0.053*	
C6	0.7443 (2)	0.51649 (12)	0.84514 (15)	0.0358 (10)	
H6	0.6428	0.5223	0.8342	0.043*	
C7	0.5653 (2)	0.48049 (10)	1.00308 (14)	0.0284 (9)	
C8	0.6274 (2)	0.53733 (11)	1.02723 (14)	0.0339 (9)	
H8	0.7233	0.5468	1.0154	0.041*	
C9	0.5493 (3)	0.58024 (9)	1.06870 (15)	0.0424 (10)	
H9	0.5917	0.6191	1.0852	0.051*	
C10	0.4089 (3)	0.56631 (11)	1.08601 (15)	0.0433 (12)	
H10	0.3555	0.5956	1.1144	0.052*	
C11	0.3468 (2)	0.50947 (13)	1.06186 (16)	0.0407 (11)	
H11	0.2509	0.4999	1.0737	0.049*	
C12	0.4249 (2)	0.46656 (10)	1.02039 (16)	0.0330 (9)	
H12	0.3825	0.4277	1.0039	0.040*	
C13	0.6284 (4)	0.36230 (18)	0.7687 (2)	0.0311 (9)	
H13A	0.5700	0.3395	0.7261	0.047*	
H13B	0.6998	0.3340	0.7963	0.047*	
H13C	0.6784	0.3969	0.7445	0.047*	
C14	0.3937 (4)	0.45715 (19)	0.7988 (2)	0.0378 (10)	
H14A	0.3678	0.4848	0.8424	0.057*	
H14B	0.3055	0.4396	0.7706	0.057*	
H14C	0.4463	0.4810	0.7604	0.057*	
C15	0.2877 (4)	0.3024 (2)	1.0248 (3)	0.0391 (10)	
H15A	0.2035	0.3285	1.0080	0.059*	
H15B	0.3546	0.3257	1.0628	0.059*	
H15C	0.2557	0.2645	1.0512	0.059*	
C16	0.2766 (4)	0.2188 (2)	0.8767 (3)	0.0420 (11)	
H16A	0.3063	0.2172	0.8215	0.063*	
H16B	0.1730	0.2283	0.8748	0.063*	
H16C	0.2956	0.1784	0.9033	0.063*	
C17	0.5663 (3)	0.19242 (10)	1.05885 (13)	0.0303 (9)	
C18	0.5334 (3)	0.13181 (12)	1.03342 (11)	0.0312 (9)	
H18	0.5345	0.1210	0.9779	0.037*	
C19	0.4989 (3)	0.08701 (9)	1.08926 (16)	0.0406 (11)	
H19	0.4764	0.0456	1.0719	0.049*	
C20	0.4973 (3)	0.10283 (12)	1.17053 (14)	0.0457 (11)	
H20	0.4737	0.0722	1.2087	0.055*	
C21	0.5302 (3)	0.16344 (14)	1.19597 (11)	0.0542 (12)	
H21	0.5291	0.1742	1.2515	0.065*	
C22	0.5646 (3)	0.20824 (10)	1.14013 (15)	0.0443 (11)	
H22	0.5871	0.2497	1.1575	0.053*	
C23	0.6851 (2)	0.20647 (11)	0.90486 (12)	0.0255 (9)	
C24	0.8044 (2)	0.16907 (12)	0.92776 (11)	0.0345 (9)	
H24	0.8388	0.1655	0.9833	0.041*	
C25	0.8732 (2)	0.13685 (11)	0.86935 (16)	0.0436 (11)	
H25	0.9547	0.1113	0.8850	0.052*	
C26	0.8228 (3)	0.14204 (12)	0.78803 (14)	0.0438 (11)	
H26	0.8698	0.1200	0.7481	0.053*	
C27	0.7035 (3)	0.17944 (12)	0.76513 (10)	0.0371 (10)	
H27	0.6691	0.1830	0.7096	0.045*	
C28	0.6347 (2)	0.21165 (11)	0.82354 (13)	0.0310 (9)	
H28	0.5532	0.2372	0.8079	0.037*	
C29	0.9060 (4)	0.39081 (19)	1.0826 (2)	0.0300 (9)	
C30	0.8865 (4)	0.31736 (18)	0.9509 (2)	0.0273 (9)	
C31	0.8607 (4)	0.27757 (19)	1.0996 (2)	0.0337 (9)	
C32	0.6494 (4)	0.36119 (18)	1.1135 (2)	0.0284 (9)	
Cr1	0.76936 (6)	0.33701 (3)	1.03423 (4)	0.02284 (14)	
O1	0.4032 (2)	0.34064 (12)	0.87633 (15)	0.0311 (6)	
O2	0.9939 (3)	0.42194 (14)	1.11716 (16)	0.0440 (8)	
O3	0.9613 (3)	0.30592 (13)	0.90084 (16)	0.0384 (7)	
O4	0.9272 (3)	0.24106 (14)	1.14056 (18)	0.0548 (9)	
O5	0.5824 (3)	0.37908 (14)	1.16462 (17)	0.0469 (8)	
P1	0.66695 (9)	0.42154 (5)	0.95043 (6)	0.0236 (2)	
P2	0.60852 (10)	0.25311 (5)	0.98419 (6)	0.0247 (2)	
Si1	0.50981 (10)	0.39322 (5)	0.84235 (6)	0.0283 (3)	
Si2	0.38082 (10)	0.28057 (5)	0.93478 (6)	0.0288 (3)	

### Atomic displacement parameters (Å^2^)

**Table:**

	*U*^11^	*U*^22^	*U*^33^	*U*^12^	*U*^13^	*U*^23^
C1	0.038 (2)	0.021 (2)	0.019 (2)	0.0002 (17)	0.0021 (16)	−0.0045 (16)
C2	0.036 (2)	0.035 (3)	0.026 (2)	−0.0021 (19)	0.0065 (17)	0.0034 (19)
C3	0.038 (2)	0.049 (3)	0.035 (3)	−0.009 (2)	0.0054 (19)	−0.002 (2)
C4	0.069 (3)	0.043 (3)	0.029 (2)	−0.021 (2)	0.015 (2)	0.004 (2)
C5	0.063 (3)	0.033 (3)	0.036 (3)	−0.004 (2)	−0.003 (2)	0.007 (2)
C6	0.044 (2)	0.030 (2)	0.032 (2)	0.003 (2)	−0.0028 (19)	0.0019 (19)
C7	0.033 (2)	0.031 (2)	0.020 (2)	0.0103 (18)	−0.0037 (16)	0.0010 (17)
C8	0.038 (2)	0.035 (2)	0.027 (2)	0.0046 (19)	−0.0038 (17)	−0.0067 (19)
C9	0.056 (3)	0.041 (3)	0.029 (2)	0.009 (2)	−0.004 (2)	−0.012 (2)
C10	0.059 (3)	0.051 (3)	0.019 (2)	0.029 (2)	−0.001 (2)	−0.003 (2)
C11	0.040 (2)	0.051 (3)	0.031 (2)	0.010 (2)	0.0019 (19)	−0.003 (2)
C12	0.036 (2)	0.032 (2)	0.032 (2)	0.0053 (18)	0.0060 (18)	−0.0047 (19)
C13	0.037 (2)	0.034 (2)	0.021 (2)	0.0009 (18)	−0.0046 (17)	0.0011 (17)
C14	0.032 (2)	0.043 (3)	0.036 (2)	0.0092 (19)	−0.0067 (18)	0.001 (2)
C15	0.027 (2)	0.048 (3)	0.043 (3)	−0.003 (2)	0.0073 (18)	−0.009 (2)
C16	0.028 (2)	0.043 (3)	0.055 (3)	−0.004 (2)	0.0000 (19)	−0.012 (2)
C17	0.027 (2)	0.032 (2)	0.031 (2)	−0.0007 (18)	0.0014 (17)	0.0025 (18)
C18	0.030 (2)	0.034 (2)	0.029 (2)	−0.0039 (18)	0.0021 (17)	0.0017 (18)
C19	0.034 (2)	0.041 (3)	0.047 (3)	−0.004 (2)	0.0019 (19)	0.011 (2)
C20	0.041 (2)	0.051 (3)	0.045 (3)	−0.014 (2)	0.006 (2)	0.018 (2)
C21	0.074 (3)	0.057 (3)	0.033 (3)	−0.012 (3)	0.014 (2)	0.005 (2)
C22	0.061 (3)	0.041 (3)	0.032 (3)	−0.012 (2)	0.011 (2)	−0.001 (2)
C23	0.026 (2)	0.022 (2)	0.029 (2)	−0.0021 (16)	0.0024 (16)	−0.0007 (16)
C24	0.038 (2)	0.032 (2)	0.034 (2)	0.0066 (19)	0.0033 (17)	−0.0038 (19)
C25	0.039 (2)	0.040 (3)	0.052 (3)	0.010 (2)	0.009 (2)	−0.004 (2)
C26	0.049 (3)	0.039 (3)	0.047 (3)	−0.004 (2)	0.024 (2)	−0.012 (2)
C27	0.046 (2)	0.038 (3)	0.028 (2)	−0.002 (2)	0.0095 (19)	−0.0052 (19)
C28	0.035 (2)	0.031 (2)	0.027 (2)	−0.0007 (18)	0.0044 (17)	0.0023 (18)
C29	0.035 (2)	0.032 (2)	0.023 (2)	0.004 (2)	0.0045 (17)	0.0058 (18)
C30	0.025 (2)	0.026 (2)	0.030 (2)	0.0028 (17)	−0.0065 (18)	0.0000 (18)
C31	0.042 (2)	0.032 (2)	0.026 (2)	−0.003 (2)	−0.0026 (18)	−0.001 (2)
C32	0.032 (2)	0.027 (2)	0.025 (2)	−0.0029 (19)	−0.0030 (19)	0.0018 (18)
Cr1	0.0245 (3)	0.0260 (3)	0.0177 (3)	0.0008 (3)	0.0004 (2)	0.0006 (3)
O1	0.0225 (13)	0.0387 (16)	0.0310 (15)	0.0027 (13)	−0.0036 (11)	−0.0031 (13)
O2	0.0440 (17)	0.0467 (19)	0.0393 (18)	−0.0202 (15)	−0.0076 (14)	−0.0015 (15)
O3	0.0333 (15)	0.0500 (19)	0.0332 (17)	0.0070 (13)	0.0097 (13)	−0.0023 (14)
O4	0.066 (2)	0.047 (2)	0.047 (2)	0.0058 (16)	−0.0199 (16)	0.0163 (16)
O5	0.0479 (18)	0.067 (2)	0.0275 (17)	0.0013 (16)	0.0121 (14)	−0.0112 (16)
P1	0.0252 (5)	0.0252 (5)	0.0201 (5)	0.0034 (5)	0.0005 (4)	−0.0011 (4)
P2	0.0271 (5)	0.0257 (5)	0.0213 (5)	−0.0002 (5)	0.0032 (4)	−0.0007 (4)
Si1	0.0296 (6)	0.0309 (6)	0.0234 (6)	0.0046 (5)	−0.0030 (5)	−0.0015 (5)
Si2	0.0241 (5)	0.0326 (6)	0.0298 (6)	0.0001 (5)	0.0023 (5)	−0.0048 (5)

### Geometric parameters (Å, °)

**Table:**

C1—C2	1.3900	C16—H16C	0.9800
C1—C6	1.3900	C17—C18	1.3900
C1—P1	1.860 (2)	C17—C22	1.3900
C2—C3	1.3900	C17—P2	1.860 (2)
C2—H2	0.9500	C18—C19	1.3900
C3—C4	1.3900	C18—H18	0.9500
C3—H3	0.9500	C19—C20	1.3900
C4—C5	1.3900	C19—H19	0.9500
C4—H4	0.9500	C20—C21	1.3900
C5—C6	1.3900	C20—H20	0.9500
C5—H5	0.9500	C21—C22	1.3900
C6—H6	0.9500	C21—H21	0.9500
C7—C8	1.3900	C22—H22	0.9500
C7—C12	1.3900	C23—C24	1.3900
C7—P1	1.8419 (19)	C23—C28	1.3900
C8—C9	1.3900	C23—P2	1.8437 (19)
C8—H8	0.9500	C24—C25	1.3900
C9—C10	1.3900	C24—H24	0.9500
C9—H9	0.9500	C25—C26	1.3900
C10—C11	1.3900	C25—H25	0.9500
C10—H10	0.9500	C26—C27	1.3900
C11—C12	1.3900	C26—H26	0.9500
C11—H11	0.9500	C27—C28	1.3900
C12—H12	0.9500	C27—H27	0.9500
C13—Si1	1.838 (4)	C28—H28	0.9500
C13—H13A	0.9800	C29—O2	1.163 (4)
C13—H13B	0.9800	C29—Cr1	1.841 (4)
C13—H13C	0.9800	C30—O3	1.155 (4)
C14—Si1	1.847 (4)	C30—Cr1	1.881 (4)
C14—H14A	0.9800	C31—O4	1.173 (4)
C14—H14B	0.9800	C31—Cr1	1.829 (4)
C14—H14C	0.9800	C32—O5	1.161 (4)
C15—Si2	1.849 (4)	C32—Cr1	1.869 (4)
C15—H15A	0.9800	Cr1—P1	2.4223 (11)
C15—H15B	0.9800	Cr1—P2	2.4322 (11)
C15—H15C	0.9800	O1—Si1	1.632 (3)
C16—Si2	1.854 (4)	O1—Si2	1.635 (3)
C16—H16A	0.9800	P1—Si1	2.2856 (13)
C16—H16B	0.9800	P2—Si2	2.2716 (13)
			
C2—C1—C6	120.0	C21—C20—H20	120.0
C2—C1—P1	120.76 (14)	C19—C20—H20	120.0
C6—C1—P1	119.24 (14)	C20—C21—C22	120.0
C1—C2—C3	120.0	C20—C21—H21	120.0
C1—C2—H2	120.0	C22—C21—H21	120.0
C3—C2—H2	120.0	C21—C22—C17	120.0
C4—C3—C2	120.0	C21—C22—H22	120.0
C4—C3—H3	120.0	C17—C22—H22	120.0
C2—C3—H3	120.0	C24—C23—C28	120.0
C3—C4—C5	120.0	C24—C23—P2	117.76 (13)
C3—C4—H4	120.0	C28—C23—P2	122.03 (13)
C5—C4—H4	120.0	C23—C24—C25	120.0
C6—C5—C4	120.0	C23—C24—H24	120.0
C6—C5—H5	120.0	C25—C24—H24	120.0
C4—C5—H5	120.0	C24—C25—C26	120.0
C5—C6—C1	120.0	C24—C25—H25	120.0
C5—C6—H6	120.0	C26—C25—H25	120.0
C1—C6—H6	120.0	C27—C26—C25	120.0
C8—C7—C12	120.0	C27—C26—H26	120.0
C8—C7—P1	121.32 (14)	C25—C26—H26	120.0
C12—C7—P1	118.66 (14)	C26—C27—C28	120.0
C7—C8—C9	120.0	C26—C27—H27	120.0
C7—C8—H8	120.0	C28—C27—H27	120.0
C9—C8—H8	120.0	C27—C28—C23	120.0
C10—C9—C8	120.0	C27—C28—H28	120.0
C10—C9—H9	120.0	C23—C28—H28	120.0
C8—C9—H9	120.0	O2—C29—Cr1	175.5 (3)
C9—C10—C11	120.0	O3—C30—Cr1	178.3 (3)
C9—C10—H10	120.0	O4—C31—Cr1	175.9 (4)
C11—C10—H10	120.0	O5—C32—Cr1	175.3 (3)
C12—C11—C10	120.0	C31—Cr1—C29	85.09 (17)
C12—C11—H11	120.0	C31—Cr1—C32	92.86 (17)
C10—C11—H11	120.0	C29—Cr1—C32	87.19 (16)
C11—C12—C7	120.0	C31—Cr1—C30	90.68 (17)
C11—C12—H12	120.0	C29—Cr1—C30	92.24 (16)
C7—C12—H12	120.0	C32—Cr1—C30	176.35 (18)
Si1—C13—H13A	109.5	C31—Cr1—P1	174.94 (13)
Si1—C13—H13B	109.5	C29—Cr1—P1	90.02 (12)
H13A—C13—H13B	109.5	C32—Cr1—P1	88.19 (12)
Si1—C13—H13C	109.5	C30—Cr1—P1	88.20 (12)
H13A—C13—H13C	109.5	C31—Cr1—P2	85.67 (13)
H13B—C13—H13C	109.5	C29—Cr1—P2	170.75 (12)
Si1—C14—H14A	109.5	C32—Cr1—P2	93.36 (12)
Si1—C14—H14B	109.5	C30—Cr1—P2	87.78 (12)
H14A—C14—H14B	109.5	P1—Cr1—P2	99.22 (4)
Si1—C14—H14C	109.5	Si1—O1—Si2	149.42 (16)
H14A—C14—H14C	109.5	C7—P1—C1	101.72 (12)
H14B—C14—H14C	109.5	C7—P1—Si1	103.63 (9)
Si2—C15—H15A	109.5	C1—P1—Si1	101.09 (9)
Si2—C15—H15B	109.5	C7—P1—Cr1	115.56 (9)
H15A—C15—H15B	109.5	C1—P1—Cr1	116.54 (8)
Si2—C15—H15C	109.5	Si1—P1—Cr1	116.09 (5)
H15A—C15—H15C	109.5	C23—P2—C17	102.33 (12)
H15B—C15—H15C	109.5	C23—P2—Si2	106.78 (9)
Si2—C16—H16A	109.5	C17—P2—Si2	100.11 (9)
Si2—C16—H16B	109.5	C23—P2—Cr1	112.29 (9)
H16A—C16—H16B	109.5	C17—P2—Cr1	116.68 (9)
Si2—C16—H16C	109.5	Si2—P2—Cr1	116.92 (5)
H16A—C16—H16C	109.5	O1—Si1—C13	113.43 (16)
H16B—C16—H16C	109.5	O1—Si1—C14	107.24 (16)
C18—C17—C22	120.0	C13—Si1—C14	111.74 (18)
C18—C17—P2	120.29 (14)	O1—Si1—P1	106.01 (10)
C22—C17—P2	119.69 (14)	C13—Si1—P1	103.80 (12)
C19—C18—C17	120.0	C14—Si1—P1	114.63 (14)
C19—C18—H18	120.0	O1—Si2—C15	111.88 (17)
C17—C18—H18	120.0	O1—Si2—C16	110.00 (17)
C18—C19—C20	120.0	C15—Si2—C16	109.82 (19)
C18—C19—H19	120.0	O1—Si2—P2	104.67 (10)
C20—C19—H19	120.0	C15—Si2—P2	105.15 (14)
C21—C20—C19	120.0	C16—Si2—P2	115.21 (14)
